# Editorial: Plant responses to abiotic stress: unraveling complex mechanisms through genomics and physiology

**DOI:** 10.3389/fpls.2026.1888934

**Published:** 2026-06-22

**Authors:** Mizanur Rahman, Takashi Asaeda, Md Harun Rashid

**Affiliations:** 1 School of Earth, Environmental and Marine Sciences, University of Texas Rio Grande Valley, Brownsville, TX, United States; 2 Graduate School of Science and Engineering, Saitama University, Saitama, Japan; 3 Hydro Technology Institute, Tokyo, Japan; 4 Research and Development Center, Nippon Koei, Tsukuba, Japan; 5 Department of Agronomy, Bangladesh Agricultural University, Mymensingh, Bangladesh

**Keywords:** abiotic stress, multi-omics integration, oxidative stress, plant stress physiology, redox homeostasis, stress-responsive signaling pathway

Abiotic stresses such as drought, salinity, alkalinity flooding nutrient deficiency and heat stress remain one of the largest concerns to assure plant production ecosystem stability and global food security. As climatic circumstances become more variable, elucidating the mechanisms that govern plant sensing of stressful environments and signalling an appropriate response is a priority in plant science. This Research Topic, *“Plant Responses to Abiotic Stress: Unravelling Complex Mechanisms through Genomics and Physiology,”* brings acquire a diverse Research Topic that collectively advance our scope of plant stress responses from physiological, biochemical, molecular, genomic, transcriptomic, and structural viewpoints.

A prominent theme emerging from this Research Topic is that plant adaptation to abiotic stress depends on the coordinated regulation of redox homeostasis and oxidative stress responses. Wilfert et al. examined the effects of flooding and salinity on stress physiology in Quercus robur exposed to controlled reduced tidal systems and emphasized that employing hydrogen peroxide (H_2_O_2_) as an indicator of stress under fluctuating environmental conditions remains challenging. In the same ways, inverse connection of activity of various antioxidative enzymes (superoxide dismutase, catalase, peroxidases and enzymes involved in ascorbate–glutathione cycle) identified for sweet potato breeding lines under osmotic stress provides an insight on mechanisms underlying stress tolerance which may also constitute useful physiological markers in breeding practices (Mishra et al.). In blueberry, the genome-wide characterization of glutathione S-transferase (GST) family provided insight into detoxification mechanisms essential for adaptation under abiotic stresses including drought, aluminium and cadmium stress conditions. When taken as a whole, these outcomes indicate how important redox control and efficient reactive oxygen species (ROS) scavenging are to plant stress tolerance (Tian et al.).

Multiple contributions also confirmed the strong interplay between abiotic stress and leaves’ photosynthetic regulation. The analysis of the cranberry drought study revealed a significant impact on photosystem activity, chlorophyll stability, oxidative damage and transcriptomic regulation by water deficit illustrating extreme sensitivity of photosynthetic machinery to prolonged stress (Chen et al.). Genome-wide identification and expression analysis of the Chloroplast Protein 12 (CP12) gene family in cotton reveals roles for CP12 proteins in enhancing heat tolerance by regulation of chloroplast redox balance and sustaining Calvin–Benson–Bassham cycle function (Li et al.). Similarly, the okra rootstock study revealed an increase in drought tolerance through spectral light quality via photosynthetic performance, antioxidant activity and thylakoidal protein complexes (Suresh et al.). Cumulatively, these studies provide additional support for the role of maintenance of photosynthetic integrity as a key determinant of stress tolerance.

Hormonal signalling and stress-responsive regulatory pathways were also identified as important mediators of plant adaptation. Chen et al. showed that hybrid *Pennisetum* heat stress activates α–linolenic acid metabolism and jasmonic acid biosynthesis, and exogenous jasmonic acid promotes thermotolerance as well as the maintenance of chlorophyll content and water content under elevated temperature. In support of these observations, the review concerning Late embryogenesis abundant (LEA) proteins and abscisic acid (ABA) signalling provided another example of the interplay between ABA-mediated signalling and synthesis/accumulation of LEA proteins for stress adaption (Hao et al.). The review proposed a unifying framework in which the ABA signaling, the transcriptional activation process, osmotic adjustment response, membrane stabilization and molecular chaperone functions all synergistically act together to enhance stress tolerance. These studies provide insight into how hormonal signaling pathways coordinate the perception of environmental stress through feedback with downstream physiological and molecular events. Ion homeostasis and membrane transport under salinity stress is another major theme of this Research Topic. he activatable salt tolerance marker, the mulberry Morus notabilis Salt Overly Sensitive 3 (MnSOS3) protein was shown to regulate Na^+^/K^+^ homeostasis enhancing (Salt Overly Sensitive) SOS signalling pathway through salinity-induced plasma membrane localization (Liu et al.). Similarly, Gong et al. identified and characterized chloride channel gene family members in potato and showed that multiple (*solanum tuberosum* Chloride Channel) StCLC genes are strongly induced under salt stress, particularly in roots, suggesting important functions in chloride transport and ionic balance. In summary, these studies underscore the pivotal participation of transport systems that associate with membranes within plant adaptation to salinity.

Various molecular approaches such as transcriptomics and multi-omics also featured prominently in the studies described within this topic. In an extensive comparative transcriptomic analysis, contrasting rice genotypes were found to adopt distinct response strategies in terms of iron transport/deficiency and homeostasis under low available iron environments. In fenugreek, genome-wide transcriptome analysis revealed differentially expressed genes that were involved in flavonoid biosynthesis, hormone signal transduction pathways, antioxidant defence mechanisms and carbohydrate metabolism (Wang et al.). Similarly, integrated transcriptomic and metabolomic analyses of red-leaf cotton revealed extensive dynamic regulation of the biosynthesis of flavonoids and accumulation of anthocyanins in response to both drought and re watering at specific developmental stages with observable metabolic recovery. Together, these studies show the relevance of systems-level approaches to connect gene expression with physiologic and metabolic response to stress (Zhang et al.).

Apart from molecular and biochemical responses a few studies focus on the importance of structural and physiological adaptation. Genome-wide analysis of GDSL lipase family genes revealed the potential contribution of stomatal outer cuticular ledge development in regulating water-loss and drought tolerance in *Dendrobium catenatum*, highlighting a general procedure for adaptively modifying the epidermal cuticular features during stress conditions (Tang et al.). Moreover, investigations analysing stomatal adaptability, chloroplast arrangement and leaf histological variation confirmed that stress tolerance includes the simultaneous responses operating at cell, tissue, and plant level (Tang et al.).

**Figure 1:**
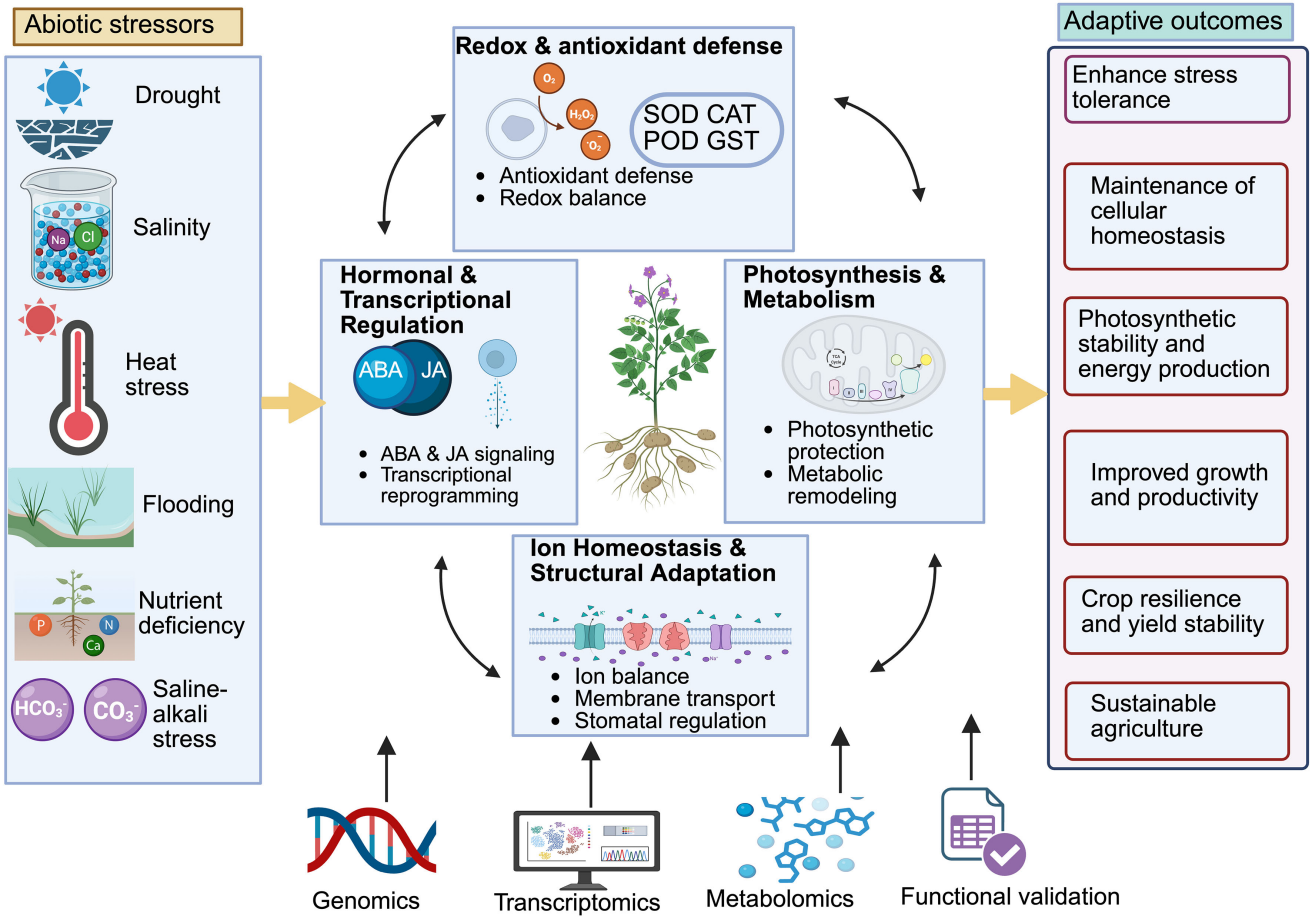
Integrated physiological and molecular mechanisms underlying plant adaptation to abiotic stress (Created in BioRender. Mizanur Rahman (2026) https://BioRender.com/5i90djl). Conceptual basis for the physiological, molecular, biochemical and structural processes by which plants adapt to abiotic stresses. Drought, salinity, flooding, heat and nutrient deficiency can each activate interconnected array of regulatory networks including those involved in redox homeostasis, hormonal signaling, ion transport, photosynthetic protection, transcriptomic reprogramming metabolic remodeling and structural adaptation to compensate for the stress., thereby determining crop resistance to climate-induced stresses

As a whole, the articles featured in this Research Topic confirm that abiotic stress tolerance in plants is a surprisingly complex and active process requiring integration of distal mechanisms including redox regulation, hormone signaling, photosynthetic protection, ion transport ability metabolic reprogramming transcriptional reprogramming and structural adaptation (**Figure 1**). The integration of physiology with genomics, transcriptomics, metabolomics, and functional validation approaches provides valuable opportunities for identifying key regulatory hubs associated with stress resilience.

Future studies should be pursued toward the integration of multi-omics technologies with field-based phenotyping, gene functional validation and precision breeding approaches in order to capture adaptive/trait responses of plants under realistic growth conditions. A larger focus should also be on identification of conserved pathways that mediate stress response to multiple abiotic stresses in eco-evolutionary circumstances while acknowledging species-and even tissue-specific adaptive mechanisms. Such integrative approaches will be essential for developing crops capable of maintaining productivity under increasingly unpredictable climatic conditions.

In conclusion, this Research Topicsheds light on plant responses to abiotic stresses and acclaimed biological plasticity of physiological, molecular or biochemical adaptation strategies. In combination with the following studies, these ensure that we have gained insight into plant mechanisms for maintaining cell homeostasis or stomatal opening under some environmental constraints while others are able to protect photosynthetic systems and coordinate ion transport pathways at cellular level or signalled together as a network of responses to stress. Such advances would lay a critical foundation for future efforts to better equipped crops and characteristics that sustain agriculture in the global climate change context.

